# Robust identification of molecular phenotypes using semi-supervised learning

**DOI:** 10.1186/s12859-019-2885-3

**Published:** 2019-05-28

**Authors:** Heinrich Roder, Carlos Oliveira, Lelia Net, Benjamin Linstid, Maxim Tsypin, Joanna Roder

**Affiliations:** Biodesix Inc, 2970 Wilderness Pl, Ste100, Boulder, CO 80301 USA

**Keywords:** Machine learning, Clustering, Molecular phenotype, Semi-supervised learning

## Abstract

**Background:**

Modern molecular profiling techniques are yielding vast amounts of data from patient samples that could be utilized with machine learning methods to provide important biological insights and improvements in patient outcomes. Unsupervised methods have been successfully used to identify molecularly-defined disease subtypes. However, these approaches do not take advantage of potential additional clinical outcome information. Supervised methods can be implemented when training classes are apparent (e.g., responders or non-responders to treatment). However, training classes can be difficult to define when assessing relative benefit of one therapy over another using gold standard clinical endpoints, since it is often not clear how much benefit each individual patient receives.

**Results:**

We introduce an iterative approach to binary classification tasks based on the simultaneous refinement of training class labels and classifiers towards self-consistency. As training labels are refined during the process, the method is well suited to cases where training class definitions are not obvious or noisy. Clinical data, including time-to-event endpoints, can be incorporated into the approach to enable the iterative refinement to identify molecular phenotypes associated with a particular clinical variable. Using synthetic data, we show how this approach can be used to increase the accuracy of identification of outcome-related phenotypes and their associated molecular attributes. Further, we demonstrate that the advantages of the method persist in real world genomic datasets, allowing the reliable identification of molecular phenotypes and estimation of their association with outcome that generalizes to validation datasets. We show that at convergence of the iterative refinement, there is a consistent incorporation of the molecular data into the classifier yielding the molecular phenotype and that this allows a robust identification of associated attributes and the underlying biological processes.

**Conclusions:**

The consistent incorporation of the structure of the molecular data into the classifier helps to minimize overfitting and facilitates not only good generalization of classification and molecular phenotypes, but also reliable identification of biologically relevant features and elucidation of underlying biological processes.

## Background

Recent progress in multiplexed molecular profiling of tissue and blood-based patient samples has yielded a plethora of information that potentially holds the key to advances in personalized medicine. Applying modern machine learning techniques to these datasets presents particular challenges, most notably the curse of dimensionality. There are nearly always many more measured attributes or features (often of the order of many thousands) than patient samples or instances (frequently only 100–200 or less). One approach to extracting useful knowledge from such data uses unsupervised learning techniques, such as hierarchical clustering, to access the underlying structure of the feature space, allowing the elucidation of different patient phenotypes [1, 2]. However, looking for structure only in the molecular feature space without making full use of other available patient data is restrictive and may be insufficient to produce answers to many clinical questions.

Regression-based approaches have been used to leverage the information contained in continuous patient attributes, most notably time-to-event (TTE) outcomes. Methods such as regularized Cox proportional hazard (PH) models [3–5], feed-forward neural network based nonlinear PH models [6], and random survival forests [7, 8] have been explored to predict a patient’s hazard, relative risk, or survival time directly using a TTE endpoint and clinical and/or molecular data. A deep-learning based approach that simultaneously also provides a personalized treatment recommendation has also been implemented [9].

Often the goal when analyzing this type of data is to identify molecular phenotypes associated with a particular patient attribute, e.g., survival. In what follows we will denote this specific attribute of interest as the ‘endpoint’, to allow the distinction between this single attribute, for which we want to identify an associated molecular phenotype, and the many attributes in the molecular data that can be used to define that endpoint-related phenotype. One basic approach to discover an endpoint-related phenotype is to adopt supervised learning with training class labels assigned based on the endpoint [10, 11]. This methodology can work well when the endpoint is categorical and there are two or more clearly defined training classes (for example, subjects with cancer versus those without cancer, or patients demonstrating an objective response to therapy versus those who do not). However, in many cases, important endpoints are continuous variables. For example, the gold standard assessments of outcome are not binary, but rather TTE variables, such as overall survival (OS), progression-free survival, or recurrence-free survival (RFS), and these can rarely be replaced adequately by surrogate endpoints [12–14]. Defining training class labels for binary classification from continuous endpoints brings challenges, such as how to determine what is a good or a poor outcome for a particular patient or, more critically, which patients receive more or less benefit from one therapy relative to another.

A simple and commonly used approach is to dichotomize the continuous endpoint into two classes at a percentile or landmark value. This approach has several drawbacks. It reduces the information content of the endpoint variable and requires selection of an appropriate percentile or landmark threshold. In addition, for time-to-event endpoints, patients with insufficient follow up may not be able to be categorized unambiguously as achieving the landmark value. However, this approach is very likely to produce training class labels with some correlation with the unknown endpoint-related, molecularly-defined phenotype. Considering survival as the endpoint of interest, two classes can be defined by dividing the cohort at median survival. More patients in a molecularly-defined good prognosis group are likely to be in the class with survival above the median, and more patients in a molecularly-defined poor prognosis group in the class with survival below the median. Since many factors outside of the molecular data combine to impact outcomes, these training class assignments will not be perfectly correlated with the molecular survival-related phenotype. Even in the case of highly clinically relevant, biologically-identified, univariate biomarkers, such as HER2 status [15], there are patients who exhibit short TTE outcomes in the good prognosis biomarker group (e.g., HER2- patients with poor outcomes) and patients who exhibit long TTE outcomes in the poor prognosis biomarker group (e.g., HER2+ patients with good outcomes). So, one would never expect to find a molecularly-based classification that could cleanly divide patients into groups with non-overlapping endpoint values. Although these cutoff-based approximations for training class labels are not perfect, they may still be good enough to allow the discovery of endpoint-related molecular phenotypes. However, depending on the difficulty of the associated classification problem, use of a poor approximation for training class labels can lead to reduced performance of the resulting classifier and identification of a molecular phenotype with weaker association with the endpoint. This may occur either directly from errors in training class labels or indirectly if a suboptimal selection of features used in classification is made based on a poor choice of the class labels.

To access the underlying structure of the molecular feature space and allow incorporation of endpoint data when accurate training class labels are not clear, we have developed a method of classification that is based on simultaneous iterative refinement of the training class labels and the classifier model towards self-consistency (i.e., the sample training class labels are the classifications produced by the classifier). This not only yields a classifier, or test, that is able to stratify subjects into one of two endpoint-related classes, but also defines the associated molecular phenotypes. The aim is to create a self-consistent system of binary classifier and class definitions. Briefly, each sample in the development set is assigned to one of two training classes. Using these class labels and a chosen classification algorithm, a first classifier is constructed. This first classifier can then be used to stratify the development set samples, yielding sample classifications that are then used as new class labels for a second iteration of classifier development. This process is repeated until convergence to produce a final classifier which reproduces the class labels for the samples from which it is constructed. Full details are given in the Methods section.

In the case of randomly selected initial training class labels and no use of endpoint data, the method is unsupervised in nature and is analogous to clustering. When endpoint data is used to set initial training class label assignments or to steer the classifier development step (e.g., by using it to filter or prune) within the iterative refinement process, the method becomes semi-supervised. The iterative process means that the initial assignment of training class labels is no longer as critical for final classifier performance, as it is refined iteration by iteration. Hence the method is well-suited to use with TTE outcomes or in other settings where training class labels cannot be easily defined or may contain errors. The approach depends on the classifier producing unbiased classifications for instances used in its development but is otherwise agnostic to classifier development scheme. The endpoint information can be utilized to define the training class labels that initialize the iterative process or to guide the development of the classifier itself. For some classifier paradigms the latter can be achieved by directly influencing the classifier development step within each iteration of class label and classifier refinement. Henceforth we will refer to a single step of the refinement process - taking the training class labels from the previous step, constructing a new classifier using those training class labels, and producing new classifications for the development set samples using this new classifier - as one “refinement iteration”.

The iterative refinement approach (IRA) can be used to develop classification algorithms to identify binary molecular phenotypes associated with any endpoint where training class labels are not obvious. Here, we demonstrate the utility of this IRA for discovering TTE-related molecular phenotypes. In particular, we will demonstrate that the method can:

- Identify TTE-related molecular phenotypes, where training class labels are not unambiguously defined, and do this robustly, so that the strength of association between classifier-defined phenotype and TTE outcome (i.e., the effect size, as measured by the hazard ratio between the binary phenotypes) generalizes to validation sample sets;
- Identify individual molecular features associated with such TTE-related phenotypes accurately and robustly, so that sets of features identified show consistency between datasets;
- Improve detection of biological processes associated with the classifier-defined phenotypes and generalization of the these identified processes across datasets;
- Improve the reliability of the estimates of strength of association between classifier-defined phenotype and TTE outcome generated during classifier development when feature selection methods are employed.

First, we present results where synthetic data are used to construct a survival-related binary phenotype (a good survival class and a poor survival class). In this setting the true phenotype is known. Hence, it is possible to assess accuracy of identification of the true molecular phenotype via its concordance with the classifications generated by the classifiers and to evaluate accuracy of detection of the subset of features that determine the true phenotype. We show that carrying out the iterative refinement can improve identification of the true phenotype in both development and validation. In addition, we use this model system to illustrate how the approach can improve generalization of the effect size of association between survival and classifier-defined phenotype when feature selection methods are employed during classifier development.

We then show that the advantages of IRA are retained in real-world datasets, where many molecular phenotypes, with and without association with the endpoint of interest, are likely to coexist, and where these phenotypes are not known a priori. In these applications we primarily assess success in identification of TTE-related molecular phenotypes via the hazard ratio between classifier-defined groups in validation datasets and the reliability of the development set estimates of this hazard ratio. The first example uses mRNA expression data from tissue samples collected from patients with breast cancer. For this problem we also evaluate the biological processes associated with the classifier-defined phenotype using gene set enrichment analysis. Lastly, we study an mRNA expression dataset from patients with lymphoma to assess some technical aspects of the method. In this real-world data setting, we study the influence of choice of initial condition (either associated with TTE outcome or not) on the iterative process, the effect of incorporation of TTE data into classifier construction, and some convergence properties of the iterative refinement process.

## Results

### Illustration of the utility of iterative refinement approach with synthetic data

The synthetic datasets, composed of 1000 attributes, were created to contain two survival-related phenotypes, A and B. The molecular phenotypes were defined by a marked difference in mean attribute value in 100 of the 1000 attributes, which was kept constant throughout the studies. The difference in survival between phenotype A (better survival) and phenotype B (worse survival) was controlled by a parameter, α (α ≥ 0), with larger α corresponding to greater difference in survival between phenotypes and α = 0 corresponding to no difference in outcome between phenotypes. (Full details about the generation of the datasets can be found in Methods.) Multiple development datasets were generated, each containing N_S_ = 120 instances (samples). The instances were divided into the two phenotypes in the ratios 1:1, 1:3, and 3:1. Ten development set realizations were drawn at random for each N_A_:N_B_ ratio and value of α studied. Validation datasets containing 1000 samples were generated in the same manner. Using the dropout-regularized combination (DRC) classifier development approach [16–18] (see Methods and Appendix A Fig. 9), the IRA was used to attempt to identify the true phenotype within the synthetic dataset. Note that the DRC classification method incorporates ensemble averaging (“bagging”) [19]. Hence, reliable classifications can be obtained from development set results using out-of-bag estimators [20]. This is essential for the IRA to function. Initial training class labels were chosen by dichotomizing the survival times at the median.

#### Identification of the true phenotype

The concordance of the median-dichotomized initial training class labels with the true phenotype increases with α. Simulation of the sampling distribution of datasets with α = 2 and N_A_ = N_B_ = 60, showed that the median concordance was 0.62, with interquartile range (IQR) 0.58–0.65, and concordance of 0.5 was the 0.7th percentile. The median hazard ratio between true phenotypes was determined to be 1.82. Given the clear distinction between the true phenotypes in multiple attributes, this level of concordance was sufficient for the true phenotype to be identified accurately from the dichotomized training class labels for three of the realizations studied. However, six of the realizations required one additional refinement iteration to discover the true phenotype exactly, and one realization required two additional refinement iterations.

**Fig. 1:**
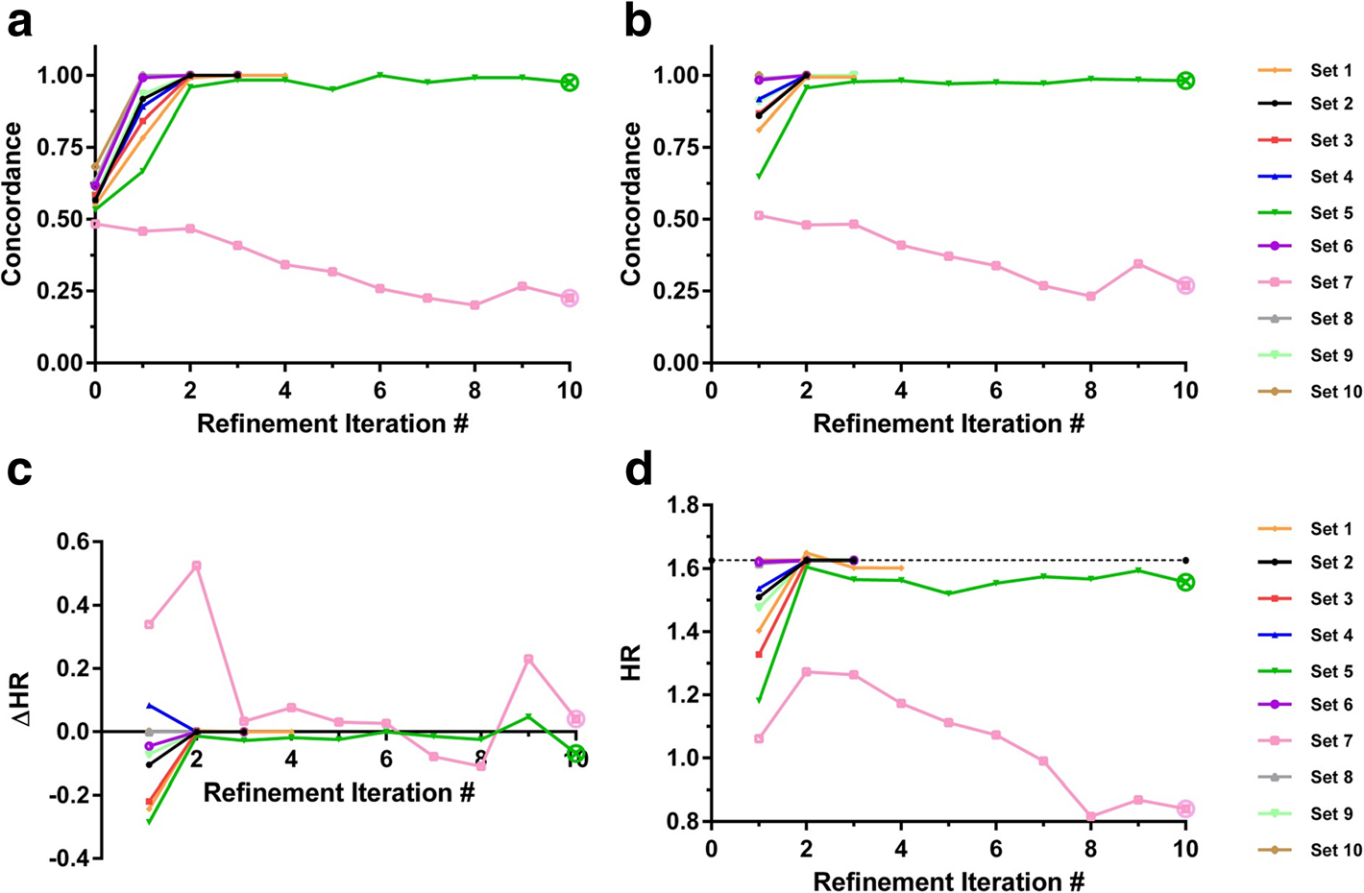
Performance of the iterative refinement approach on synthetic data with α = 1 for N_A_ = N_B_ = 60. For each development set realization the IRA was applied. At each refinement iteration, the classifiers were applied to their development set realization and the independent validation set. Concordance of classifier-derived phenotype with true phenotype is shown for (**a**) the ten development set realizations, and (**b**) the validation set, for all ten development set realizations as a function of refinement iteration. The difference between the hazard ratio for classifier-derived phenotypes and the hazard ratio for phenotype A vs phenotype B in the development sets, ∆HR, is shown in (**c**) as a function of refinement iteration. The hazard ratios for classifier-derived phenotypes in the validation set as a function of refinement iteration are shown in (**d**). The value of the hazard ratio in the validation set for phenotype A vs B (HR = 1.63) is indicated by the dashed line. The crossed open circle indicates lack of convergence after ten refinement iterations

As α is decreased to 1, concordance of the median-dichotomized training class labels with true phenotype is reduced. Sampling distribution simulations for N_A_ = N_B_ = 60 and α = 1 demonstrated median (IQR) concordance of 0.57 (0.53–0.60) with concordance of 0.5 at the 5.8th percentile. The median HR between true phenotypes was 1.43. The classifications generated from the initial training class labels in this case accurately reproduced the true phenotype in only two of the ten development set realizations. However, as shown in Fig. 1a and c, the IRA usually converged quickly and, at convergence, the classifier-defined phenotype was either identical or very close to the true phenotype. Note that for development set realization 7, for which the initial training class labels were concordant with the true phenotype for less than 50% of instances, the approach was unable to identify the true phenotype even after ten refinement iterations. The results were similar when validated in the independent set (Fig. 1b and d). The classifier built using the initial training class labels failed to identify the true phenotype with an accuracy exceeding 0.90 when constructed with five of the ten development set realizations, and only achieved an accuracy greater than 0.95 for three development set realizations. However, using the IRA, convergence and concordance greater than 99% was achieved in nine of the ten development set realizations. This improvement as a function of refinement iteration was also apparent in the HRs between classifier-derived phenotypes. Using only the initial training class labels for classifier development, the HRs achieved varied widely around that for phenotype A vs phenotype B in the development sets (Fig. 1c). In validation the classification groups generated from the initial training class labels showed smaller effect sizes than those for the true phenotypes, except for the one case where the IRA converged in one refinement iteration. For eight of the nine remaining cases the HRs between the classifier-defined phenotypes increased to, or close to, the HR between phenotype A and phenotype B with iterative refinement.

Qualitatively similar results were obtained for α = 1 with ratios N_A_:N_B_ of 30:90 and 90:30 (Appendix B Fig. 10 and Fig. 11), illustrating that utility is not restricted to identification of phenotypes of equal prevalence. While convergence of the IRA appeared to be somewhat slower in these cases of uneven phenotype prevalence, as the IRA adjusted to the imbalance in the true phenotype proportions, each of the ten development set realizations for both 1:3 and 3:1 ratios produced IRA convergence and accurate discovery of the true phenotype.

**Fig. 2:**
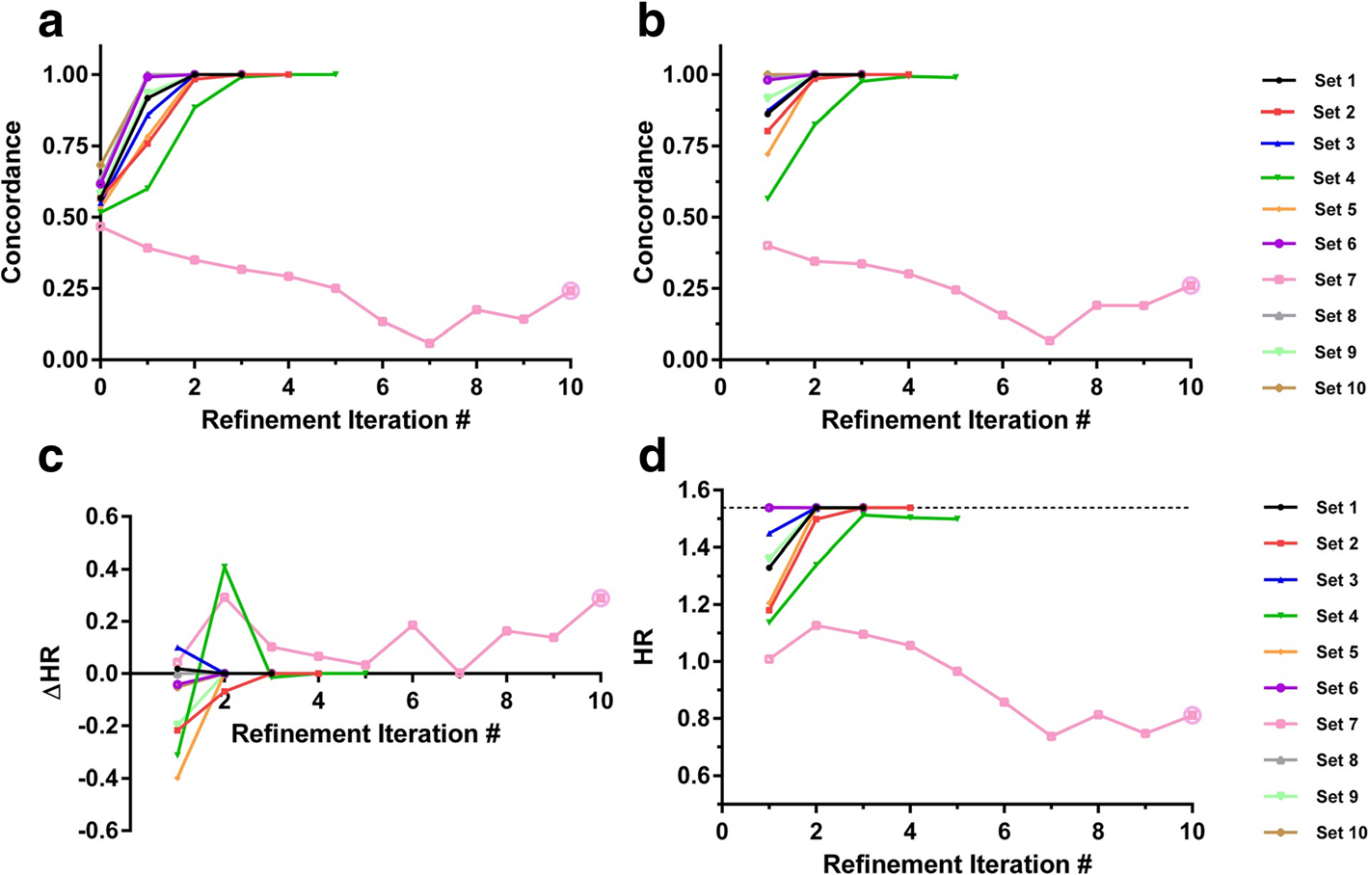
Performance of the iterative refinement approach on synthetic data with α = 0.8 for N_A_ = N_B_ = 60

Sampling distribution simulations for smaller α = 0.8 and N_A_ = N_B_ = 60 gave median (IQR) for concordance of true phenotype and dichotomized initial training class labels of 0.57 (0.53–0.58) with the 9th percentile at a concordance of 0.5; the median HR between true phenotypes was 1.35. Results from applying IRA in this setting are shown in Fig. 2. The initial training labels were not sufficiently accurate to allow discovery of the true phenotypes in all but one case (set 8). However, the IRA recovered the true phenotype with 100% accuracy for eight of the other nine development set realizations. This was mirrored in validation, where the true phenotype was detected with at least 99% accuracy by the iterative refinement process on convergence. The corresponding hazard ratios for classifier-defined phenotypes obtained using the initial class labels showed a wide range across the development set realizations and reduced effect sizes in validation. The IRA improved the HRs in the validation set in the majority of cases once convergence was achieved.

For each development set realization the IRA was applied. At each refinement iteration, the classifiers were applied to their development set realization and the independent validation set**.** Concordance of classifier-derived phenotype with true phenotype is shown for (a) the ten development set realizations, and (b) the validation set, for all ten development set realizations as a function of refinement iteration. The difference between the hazard ratio for classifier-derived phenotypes and the hazard ratio for phenotype A vs phenotype B in the development sets, ∆HR, is shown in (c) as a function of refinement iteration. The hazard ratios for classifier-derived phenotypes in the validation set as a function of refinement iteration are shown in (d). The value of the hazard ratio in the validation set for phenotype A vs B (HR = 1.54) is indicated by the dashed line. The crossed open circle indicates lack of convergence after ten refinement iterations.

**Fig. 3:**
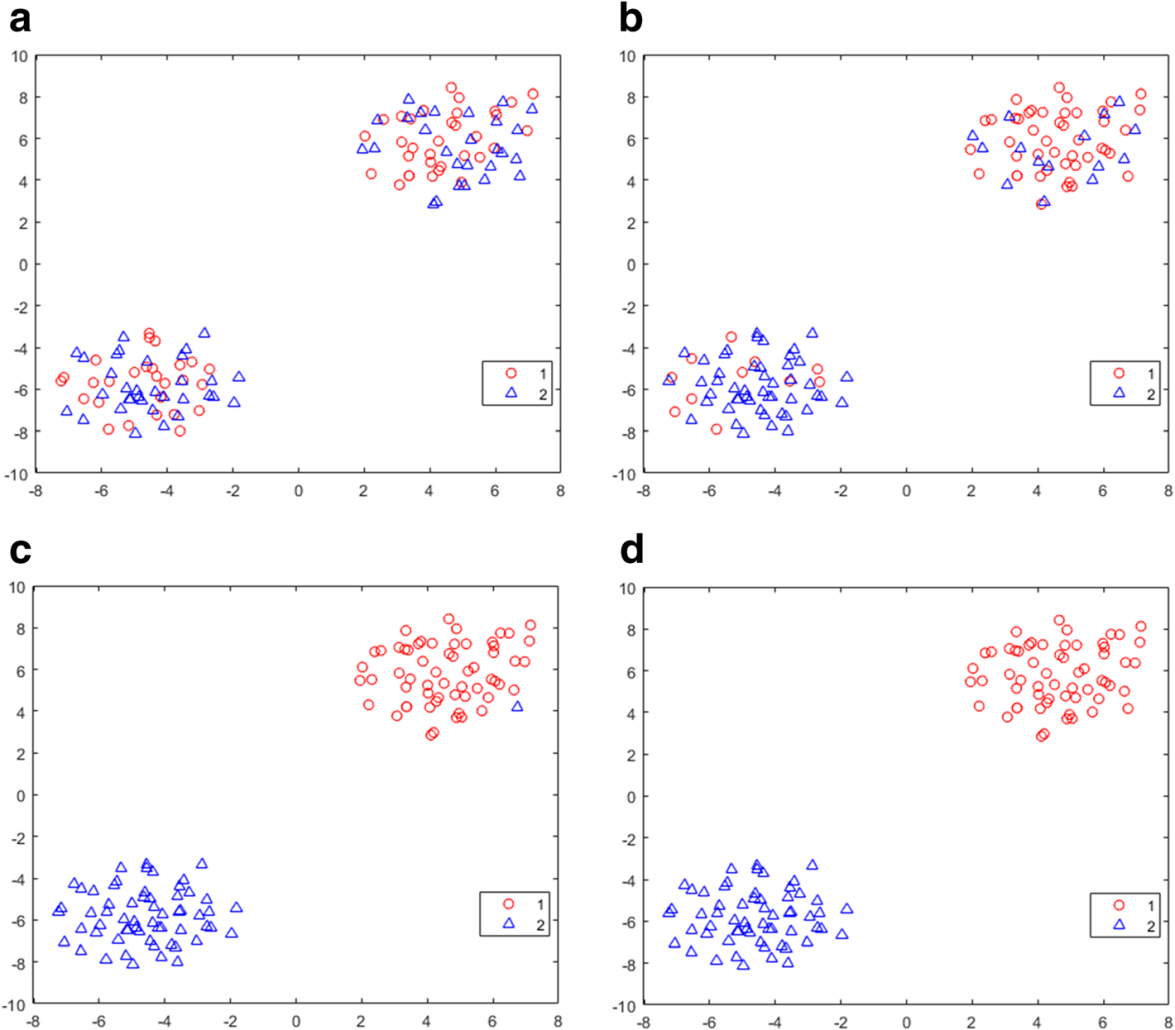
t-SNE plots for iterative refinement until convergence (α = 1, N_A_ = N_B_ = 60, development set realization 1)

The effect of the iterative refinement process was visualized within the molecular feature space using t-SNE (t-distributed stochastic neighbor embedding) plots [21]. t-SNE analysis is a tool that allows the visualization of high-dimensional data in a 2D map, capturing both local and global structure. The t-SNE plots are shown in Fig. 3 for development set realization 1 for N_A_ = N_B_ = 60 and α = 1 for each refinement iteration. The marked differences in mean value in 100 of the 1000 attributes between phenotype A and B produced two clearly separated clusters of attributes, phenotype A in the upper right of the plot and phenotype B in the lower left. There was little concordance between the initial training class labels and the underlying feature space structure (Fig. 3a), as the two training classes (1 – poor prognosis (red) and 2 – good prognosis (blue)) were spread across the two clusters. As iterative refinement occurred (Fig. 3b to Fig. 3c to Fig. 3d), the classifier-derived phenotypes changed to more closely match the molecular feature space structure. At convergence (Fig. 3d) the classifier-derived phenotype (and training labels of the classifier itself) reproduced the two compact clusters of the true phenotypes exactly. In this setting of compact regions of class labelled instances, small changes in attribute values have little impact on instance classification. Classification is then less dependent on specific details of the development set, less likely to be overfitted, and more likely to generalize well. (Corresponding t-SNE plots for the validation set classified by the classifiers developed for development set realization 1 are shown in Appendix B Fig. 12.)

Results are shown for a) initial training class labels, b) classifier-derived phenotypes using initial training class labels (refinement iteration 1), c) classifier-derived phenotypes using training class labels from refinement iteration 1 (“refinement iteration 2”), d) classifier-derived phenotypes at convergence at refinement iteration 3. x and y axes show arbitrary scales of the two t-SNE components.

#### Identification of attributes defining the true phenotypes

By construction, only 100 of the 1000 attributes are associated with the phenotype. We investigated the association of all attributes with the classifier-defined groups during the IRA. For α = 1 and α = 2, the training class labels at the second refinement iteration were always associated with the 100 true associated attributes at a Bonferroni-adjusted 95% significance level, except for one training set realization for α = 1 where the iterative refinement did not converge within 10 refinement iterations. However, these attributes were not reliably associated with the initial training class labels generated by survival dichotomization. Only one development set realization for α = 1 and one for α = 0.8 had any of the 100 attributes defining true phenotype associated with the initial training class labels at the Bonferroni-adjusted 95% significance level. One of the development set realizations (set 4) for α = 0.8 identified all 100 of the associated attributes only after the third refinement iteration.

#### Generalization of effect size during iterative refinement with feature selection

**Fig. 4:**
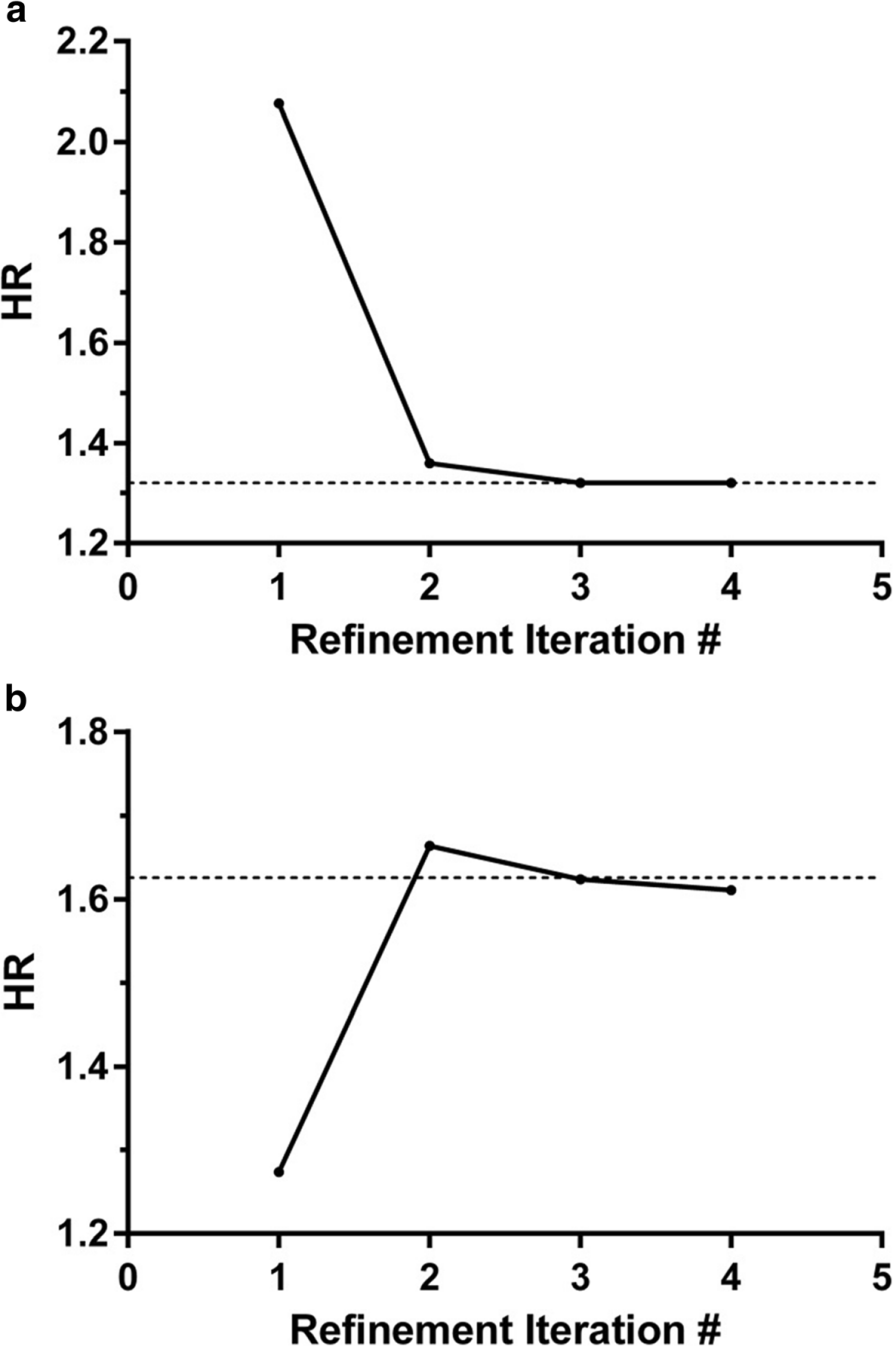
Performance of the iterative refinement approach with feature selection

Many classifier development approaches require feature selection when applied in the setting of more attributes than instances. We investigated classifier development with feature selection for the synthetic data using development set realization 1 for α = 1 and N_A_ = N_B_ = 60. At each refinement iteration, 50 of the 1000 attributes were selected based on t-test for association between training class label and survival. The results are shown in Fig. 4. The hazard ratios between classifier-defined phenotypes from the initial refinement iteration were over-estimated within the development set and the effect size was reduced in the validation set compared with that between the true phenotypes. As shown above, the attributes selected at the initial refinement iteration do not well represent those that are associated with the true phenotype. This leads to overfitting to the poorly selected attributes during classifier development and lack of generalization to and poor performance in the validation set. However, at convergence, both in the development set and in the validation set, the true phenotype was recovered to within a few percent and the corresponding HR between phenotypes was obtained. As the training class labels become more accurate during the IRA, the set of features selected converges to the set associated with the true phenotype. This allows development of robust classifiers, reliable hazard ratio estimations from the development set and good generalization to the validation set.

Classifier development was carried out selecting 50 features by t-test at each refinement iteration for α = 1 and N_A_ = N_B_ = 60 and development set realization 1. a) Hazard ratio between classifier-derived phenotypes obtained in the development set as a function of refinement iteration. The dashed line shows the HR between true phenotypes in this development set realization. b) Hazard ratio between phenotypes obtained in the validation set as a function of refinement iteration. The dashed line shows the HR between true phenotypes in the validation set.

### Real world data application 1: prognostic classifier for recurrence-free survival for patients with breast cancer treated with adjuvant chemotherapy

In this application the goal was to identify molecular phenotypes with better and worse recurrence-free survival in mRNA expression datasets from patients with breast cancer. The datasets contained 12,770 attributes each and 148, 147, and 380 patients for the development set, the internal validation set, and the independent validation set, respectively.

Using the strongly regularized bagged logistic regression classifier development method (see Methods and Appendix A for more details) we examined the process of including TTE outcome data into the classifier development by TTE-determined initial condition and TTE-based feature selection within the iterative refinement process. For each refinement iteration, the association of the training class labels with each attribute (feature) was determined by t-test. Only the 100 features with the smallest *p* values for this association (largest absolute values of t-test statistic) were selected for training the classifier within that refinement iteration. The initial condition was determined by dichotomizing recurrence-free survival (RFS), with 74 of the 148 development set samples with RFS below the median assigned to the poor prognosis group and the other samples assigned to the good prognosis group.

**Fig. 5:**
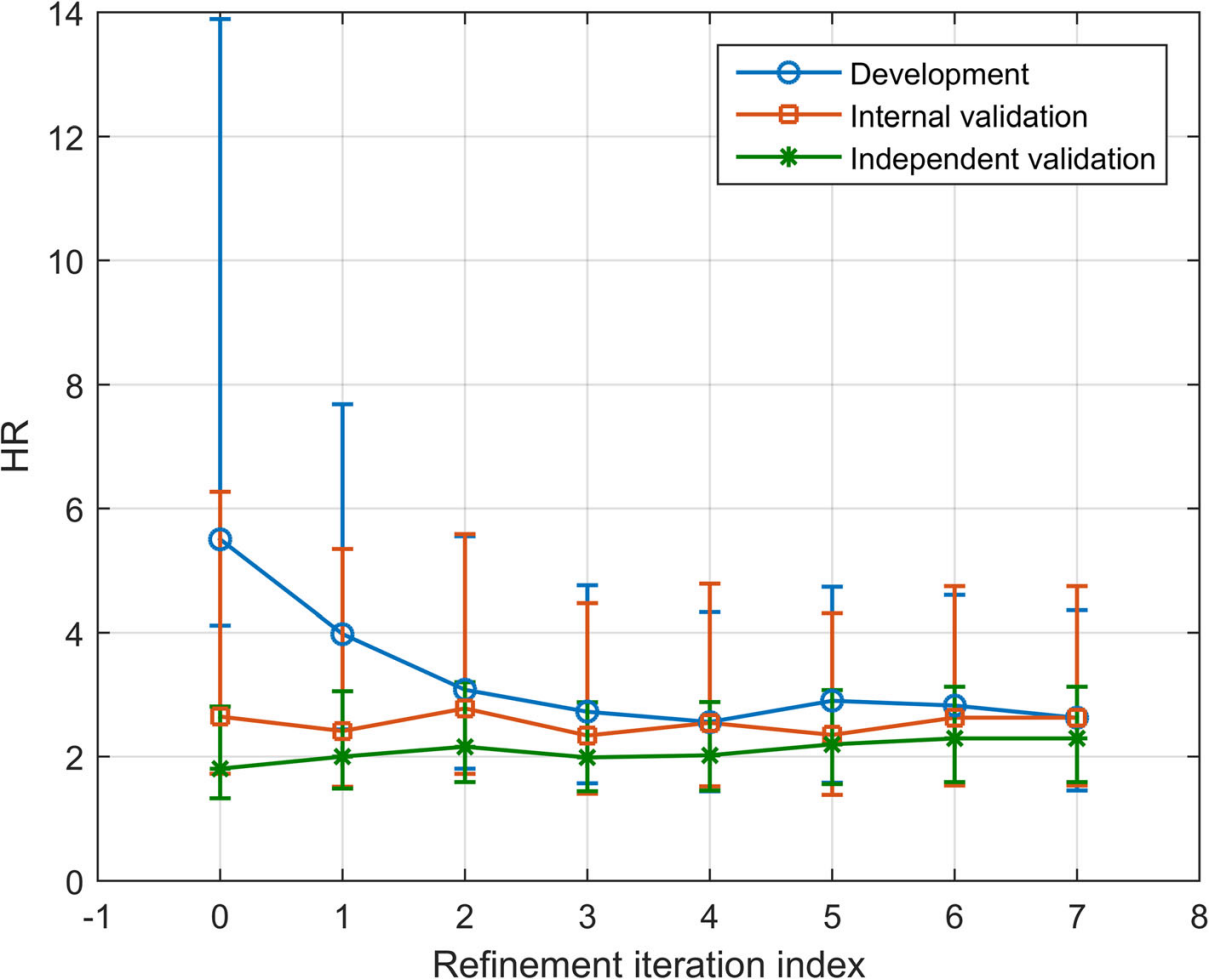
RFS HR between classifier-defined phenotypes as a function of refinement iteration

Figure 5 shows the HR between the resulting classification groups as a function of refinement iteration for out-of-bag classifications for the development set and standard classifications for the internal and independent validation sets. Initially, within the development set an overestimation of the true stratification power of the classifier occurred (HR for RFS between classification groups was 5.5 for the development set compared with 1.7 for the internal validation set). However, as the iterative refinement process continued, this overestimate of effect size diminished, and a reliable development set estimate of effect size was achieved after 3–4 refinement iterations. The lack of consistency between the TTE median-based initial training class assignments and the true feature space structure led to feature selection inconsistent with the feature space structure and to overfitting to specific details of the development set. As the IRA proceeded, the training class labels relaxed to reflect the overall feature space structure. This led to feature selection consistent with that molecular structure, with minimization of the chance for overfitting to random intricacies of associations of features and outcomes within the development set and improved generalization to the internal and independent validation sets. (This is illustrated in Appendix B Fig. 13 by t-SNE plots for the TTE median-based initial training class labels and the classifications at refinement iteration 7 for the development and validation sets.)

**Fig. 6:**
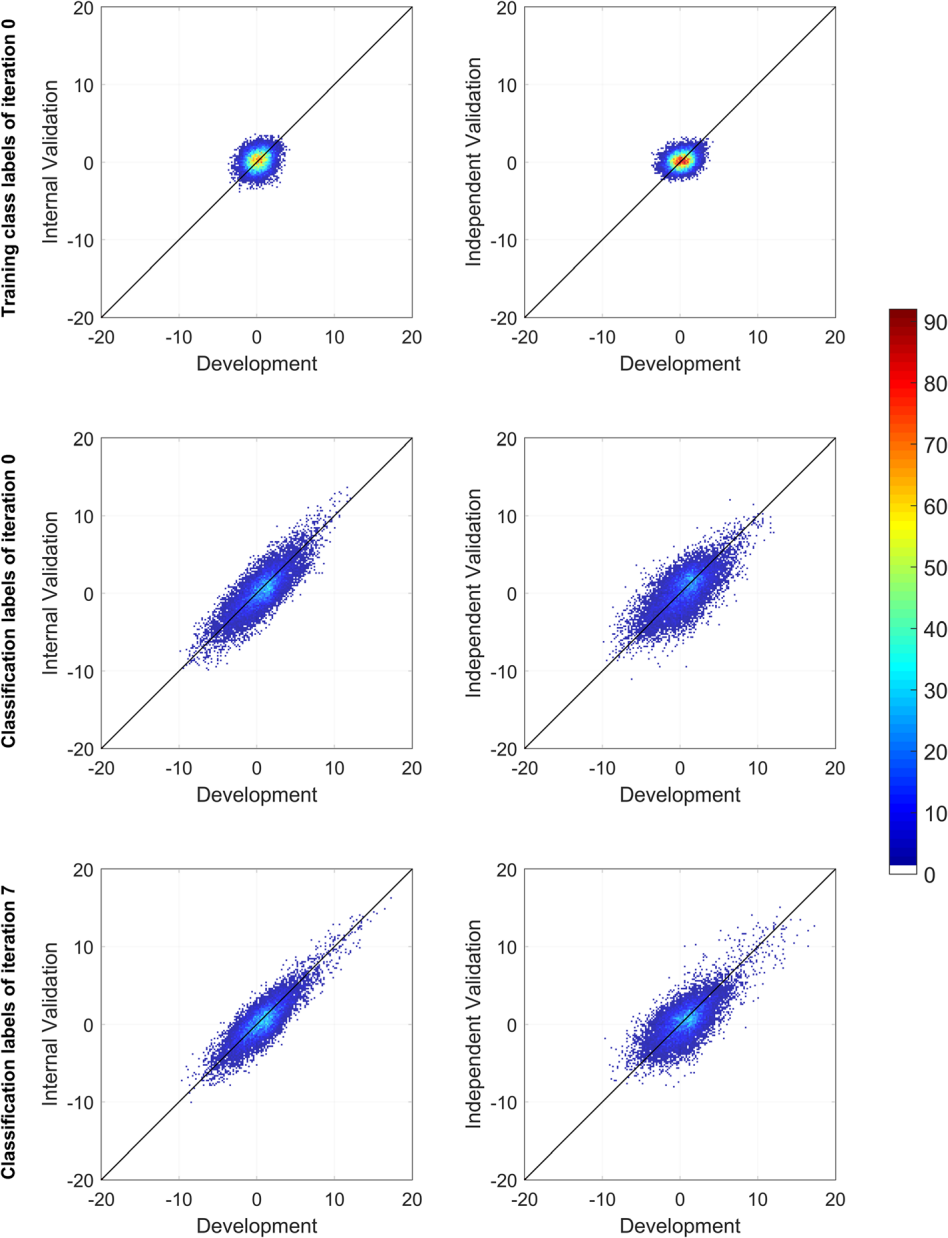
Bivariate histogram of the t-test statistics obtained for the mRNA expression attributes

This generalization extended beyond the effect size estimate of the classifier for stratification of prognosis as measured by HR to the assessment of relevance of the individual attributes for prediction of outcomes. Figure 6 compares the t-test statistics for association of each attribute with the initial training class labels and with the classifications of refinement iteration 0 and 7 between the development set and internal validation set and between the development set and independent validation set. The t-test statistics of the internal and independent validations were normalized in order to take into account the different dataset sizes by multiplying by $\sqrt{1/{N}_{1,\mathrm{val}}+1/{N}_{2,\mathrm{val}}}/\sqrt{1/{N}_{1,\mathrm{dev}}+1/{N}_{2,\mathrm{dev}}}$,  where N_1,val_ and N_2,val_ are the number of samples assigned to the poor prognosis and good prognosis classification groups in the validation set (internal or independent), and N_1,dev_ and N_2,dev_ are the number of samples assigned to the poor prognosis and good prognosis groups in the development set. There was little, if any, correlation between the features associated with the initial training class labels between the development set and either of the validation sets (Fig. 6, top row). However, coherence and consistency between the molecular feature space structure and training class assignments was achieved during the refinement process, and this generalized to the validation sets. This implies generalization of the strength of association of features with the resulting classifications.

Left panels: development and internal validation sets, right panels: development and independent validation sets. For each data set, the statistics were obtained by comparing the univariate distributions of the attributes between the two classes Poor prognosis and Good prognosis. For the top 2 panels, the labels were TTE median-based. For the medium and bottom panels, the labels were those given by the resulting classifiers of refinement iterations 0 and 7, respectively. Each panel corresponds to a bivariate histogram with 12,770 entries (number of attributes); the x- and y-axes are divided into 200 bins in the range − 20 to 20.

This can be of crucial biological importance as it means that, with this process of iterative refinement of training class labels and classifier, we can reliably access structure in the molecular data in a way that generalizes across datasets. After several refinement iterations, we can isolate the most relevant individual genes associated with the classifier-defined phenotype and these will be consistent across the datasets. For example, if we select from the 12,770 available genes, the 100 most strongly associated with the classification results of refinement iteration 7 separately for the development set, the internal validation set, and the independent validation set, we identify only 142 distinct features, 63 (44%) of which are common across all three sets.

The increased levels of association of attributes with classifier-defined phenotypes and their generalization across datasets means that the biological processes associated with the phenotypes can also be more easily and reliably detected via methods such as gene set enrichment analysis (GSEA) [22, 23]. Using software publicly available from the Broad Institute (software.broadinstitute.org/gsea/index.jsp), GSEA was carried out separately for the gene expression datasets of the development set, the internal validation set, and the independent validation set for the TTE endpoint dichotomized initial training class labels and the classifications from refinement iteration 7. The hallmark gene sets library from MSigDB was selected for the analysis [24]. Gene sets that were associated with the classifier-derived phenotypes with nominal *p* value < 0.1 and false discovery rate (FDR) q value < 0.25 were identified. Table 1 shows the gene sets identified for each dataset. With the TTE median-dichotomized training class labels, only four gene sets were identified as associated for all three datasets and nine different gene sets were identified in only one of the three datasets. For the classifications of refinement iteration 7, at convergence and consistent with the underlying feature space structure, 21 gene sets were identified as associated with the classifier-defined phenotypes in all three datasets and only five gene sets were identified in a single dataset. Hence, the phenotypes associated with the converged classifications showed clearer and more consistent associations with biological processes than those associated with the initial training class labels.

**Table 1:** Gene sets association with training class labels at iteration 0 or classification at iteration 7. Gene sets associated with *p* value < 0.1 and FDR < 0.25 in gene set enrichment analysis of the development set (DEV), the internal validation set (INT VAL) and the independent validation set (IND VAL) are marked with “X”

	Initial Training Class Labels			Classifications: Iteration 7		
Gene Set	DEV	INT VAL	IND VAL	DEV	INT VAL	IND VAL
MYC Targets V2	X	X	X	X	X	X
Spermatogenesis	X	X	X	X	X	X
G2M Checkpoint	X	X	X	X	X	X
E2F Targets	X	X	X	X	X	X
Estrogen Response Early		X	X	X	X	X
PI3K AKT MTOR Signaling		X	X	X	X	X
DNA Repair		X	X	X	X	X
Mitotic Spindle		X	X	X	X	X
MTORC1 Signaling		X	X	X	X	X
MYC Targets V1		X	X	X	X	X
Cholesterol Homeostasis		X		X	X	X
UV Response Up		X		X	X	X
Glycolysis		X		X	X	X
Unfolded Protein Response		X		X	X	X
Reactive Oxygen Species Pathway		X		X	X	X
UV Response Down		X	X	X	X	X
Oxidative Phosphorylation		X		X	X	X
Epithelial Mesenchymal Transition			X	X	X	X
Angiogenesis				X	X	X
Myogenesis				X	X	X
Coagulation				X	X	X
TGF beta Signaling			X			X
NOTCH Signaling						X
Interferon gamma Response		X		X	X	
Inflammatory Response				X		
Allograft Rejection				X		
Interferon alpha Response				X		
Estrogen Response Late			X			

There is biological rationale for many of the additional gene sets identified across all datasets using the phenotypes elucidated by the IRA. Estrogen receptor response plays a critical role in the endocrine dependence of breast cancer and the outcomes of therapy [25]. Unfolded protein response has been shown to be associated with pathogenesis of a variety of diseases [26, 27] and poor prognosis and chemotherapy resistance in breast cancer [28]. Elevation of the reactive oxygen species (ROS) pathway has been detected in almost all cancers, where it promotes many aspects of tumor development and progression [29, 30]. Dysregulation of metabolism is one of the hallmarks of cancer [31] and switching of cancer cells from oxidative phosphorylation to aerobic glycolysis as a source of energy is typical for many tumors [32]. The epithelial-mesenchymal transition is relevant for cancer prognosis as a mechanism for invasion and metastasis of cancer [33], and angiogenesis is known to be important in breast cancer, where bevacizumab, an angiogenesis inhibitor, is an approved drug.

### Real World Data Application 2: Prognostic classifier for overall survival for patients with lymphoma treated with chemotherapy

mRNA expression of 21,024 genes in samples from 181 patients with lymphoma were available for analysis, together with overall survival data. The goal was to identify binary phenotypes of patients with better and worse survival. The cohort was split into a development set (91 patients) and validation set (90 patients). Via identification of survival-related phenotypes in this dataset, we aimed to assess the following technical aspects of the IRA:

- The influence of initial training class labels on the convergence of the iterative process;
- The impact on convergence of the iterative process of tuning of the classifier development process using survival data within the IRA; and
- The relative importance of within iteration tuning of classifier development using survival data and initial training class labels on convergence of the iterative process.

For this application we used the dropout-regularized combination (DRC) classifier development method (see Methods and Appendix A) [16–18]. This is a hierarchical approach to classification in which many sub-classifiers are created from small numbers of attributes. These sub-classifiers are then applied to their training set and the hazard ratio between resulting classification groups is evaluated. Only sub-classifiers achieving a minimal level of performance on this endpoint-related metric pass filtering to be combined, regularized by dropout, into a master classifier. This is repeated for multiple splits of the development set into training and test sets, which are ensemble averaged. The filtering, or pruning, step allows for tuning of classifier performance towards improved association with the endpoint.

The IRA was implemented with four sets of initial conditions (ICs), i.e., four methods of initial training class label assignment, and three levels of filtering of the DRC sub-classifiers. The initial conditions for the training classes were selected as follows:

- Instances were randomly assigned to good or poor prognosis training classes in ten independent realizations (“random ICs”);
- Instances with overall survival (OS) below the median were assigned to the poor prognosis group and instances with OS above the median were assigned to the good prognosis group (median-dichotomized initial training class labels). Instances that could not be unambiguously assigned due to censoring were assigned half to each class in all possible combinations to yield six realizations and a seventh realization was generated by dichotomizing at the median survival time independent of censoring information (“TTE median-based ICs”);
- 10% noise in the initial class label assignments was added to the seventh realization of (b), by randomly swapping labels between pairs of samples, one in each training class, to yield ten initial training class label realizations more weakly associated with TTE outcome than (b) (“10% noise ICs”); and
- 20% noise in the initial class label assignments was added to the seventh realization of (b), by randomly swapping labels between pairs of samples, one in each training class, to yield ten initial training class label realizations more weakly associated with TTE outcome than (c) (“20% noise ICs”).

**Fig. 7:**
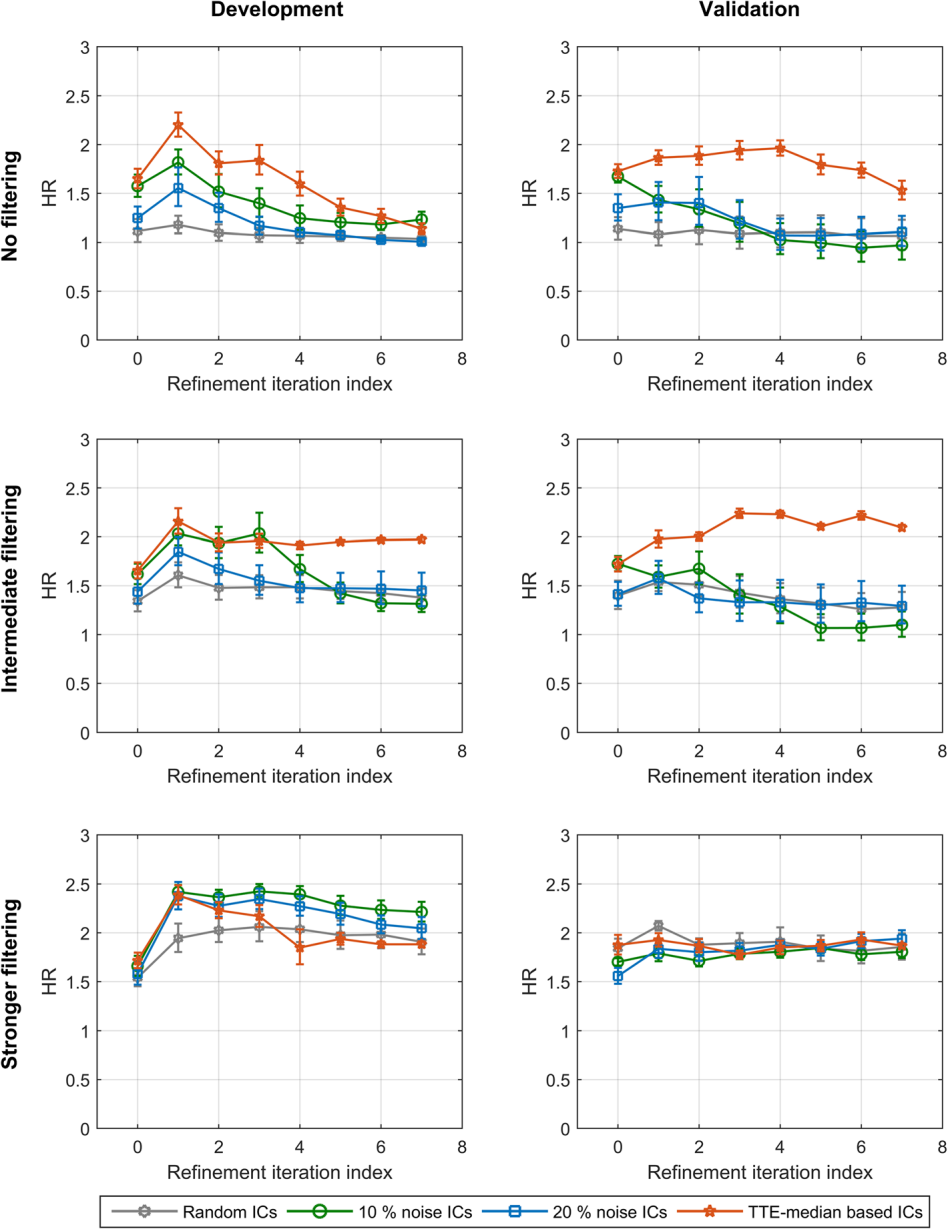
Average hazard ratio for each set of realizations of initial training class labels at each refinement iteration

Three levels of filtering of the sub-classifiers were chosen: no filtering, i.e., no pruning and all sub-classifiers combined; intermediate filtering, only sub-classifiers producing a HR for OS between classification groups of the training set between 1.3–100 accepted (around 25–35% of sub-classifiers accepted for random ICs and 25–75% accepted for other ICs, depending on refinement iteration); and stronger filtering, only sub-classifiers producing a HR for OS between classification groups of the training set between 2.0–100 accepted (around 2–10% of sub-classifiers accepted for random ICs and 5–45% for other ICs, depending on refinement iteration). The iterative refinement process was carried out for each combination of initial condition for training class labels and filtering. The results were averaged over each set of realizations of the initial training class labels. The HRs between classifier-defined phenotypes at each refinement iteration for out-of-bag classifications of the development set and standard classification of the validation set are shown in Fig. 7.

No filtering (top row), intermediate filtering (middle row) and stronger filtering (bottom row) for the development set (left) and the validation set (right). Error bars show standard error.

For random initial conditions, with no association between initial training class labels and endpoint, and no filtering, the final classifiers showed no ability to stratify patients based on OS. The average HR between classification groups over the random ICs realizations was around 1 in development and validation. Increasing to an intermediate level of filtering of sub-classifiers yielded an average HR above 1 in development and consistent results in validation. Investigation of the individual random IC realizations (Appendix B, Fig. 14) showed that two of the realizations produced validating classifiers with reasonable stratification power (HRs around 2 and 1.5), while the other eight realizations investigated produced classifiers with no prognostic utility. Further increasing the level of filtering produced classifiers with good stratification power, with average HRs between classifier-defined phenotypes around 2 in development and validation. Closer inspection of the individual ICs showed that all but one of the ten random IC realizations produced a useful classifier with the strongest filtering.

For TTE median-based ICs, it was still not possible to reliably stratify patient prognosis if no filtering was used. However, as filtering was increased, even to the intermediate level, the combination of filtering and TTE median-based initial training class assignments was sufficient to reliably produce classifiers with good performance (HRs around 2 or higher in development and validation sets).

The 10% noise and 20% noise ICs, with weaker association of initial training class labels with endpoint than for TTE median-based initial training class labels, produced classifiers similar to those for the completely random initial conditions at all three levels of filtering. For the strongest filtering all 10 and 20% noise ICs produced useful classifiers, with HRs between classifier-defined phenotypes greater than or around 1.5 in development and validation. However, only a minority or none of the noise-based ICs yielded classifiers with acceptable performance with intermediate filtering or no filtering, respectively (Appendix B Fig. 14c and d). Note that these observations at intermediate filtering illustrated that adding as little as 10% noise to initial training class label assignment can impact the ability to adequately solve the classification task.

Hence, for this particular problem of prognostic stratification in lymphoma with the DRC classifier development method, it was necessary to utilize endpoint input into both the choice of initial training class labels and filtering of the sub-classifier pool to consistently generate classifiers that could stratify patients effectively by overall survival.

Some observations could be made on the course of the IRA from comparing the convergence of the individual realizations of the four different initial training class label assignment protocols. The convergence was quite fast, with the number of changes in class label assignments between iterations dropping rapidly in the first few refinement iterations. The number of changes in the training class labels from refinement iteration 0 to 1 was typically of the order of 30% or more, while the number of changes at each refinement iteration after iteration 4, assuming no prior complete convergence, was small (typically between 1 and 4 instances). While not all realizations converged within the maximum eight refinement iterations carried out for this study, 47% did. Rate of convergence did not seem to vary by kind of initial training class assignment protocol or level of filtering, and the fixed points of the IRA could be either a definitive combination of classifier and training class assignments or a periodic attractor, e.g. a swapping back and forth of the training class labels for a pair of instances by refinement iteration. In cases where convergence was not achieved within eight refinement iterations, the number of changes in training class labels from iteration to iteration was generally small, causing small fluctuations in classifier-defined phenotype proportions. Interestingly, even with a sampling of only ten realizations, several of the random IC realizations converged to identical classifier-defined phenotypes and classifiers. This indicates that the space of fixed points for the IRA must be relatively small and certainly not of the order of the number of possible initial class label assignments.

## Discussion

We have introduced an iterative process which simultaneously refines training class labels and the associated classifier to identify endpoint-associated phenotypes in molecular datasets. At its simplest level, this approach can be viewed as an alternative clustering-like approach to discerning structure in molecular feature space which could allow us to identify patient phenotypes from the molecular data. We have shown that this iterative refinement paradigm can easily incorporate endpoint information to steer the process towards the development of classifiers suited for particular tasks. A major advantage of the process is that even when guided by endpoint data, the IRA relaxes to a self-consistency between training class label and classifier which reflects the underlying structure of the molecular feature space. This means that classifiers and classifier-defined phenotypes are relatively resistant to changes in feature values within the “clustered”, class-specific regions of feature space (see Fig. 3). Hence, phenotypes can be detected robustly, stable to the inevitable uncertainties in real-world genomic or proteomic measurements and the classifiers which identify these phenotypes can generalize well to unseen datasets. Further, this generalization can assist researchers to reliably identify genes or proteins associated with the classifier-defined phenotypes at IRA convergence. This information can be synthesized to produce more reproducible and generalizable assessments of the relevant underlying biological processes.

The IRA has been used to generate validated tests for the identification of patients likely to have good outcomes when treated with immunotherapy using high-throughput, reproducible measurements of the circulating proteome. Associated set enrichment analyses highlighted the importance of complement activation and wound healing for the classifier-defined phenotype and this novel in silico result has been confirmed by independent work using conventional clinical and preclinical methods [16, 17].

It has been recognized, particularly in the field of adjuvant breast cancer prognostic stratification, that it is possible to discover many different survival-related phenotypes using barely overlapping sets of genomic features [34]. Furthermore, Venet et al. [35] showed that most randomly selected genomic signatures identify phenotypes with similar differences in outcome between them. In practice, it may not be very relevant what genomic signature is used or precisely what phenotypes are identified, as long there is a similar difference in survival between the phenotypes, i.e., as long as the classifier or test identifying the phenotypes demonstrates similar performance and utility [36]. However, for biological understanding of the resulting phenotypes and their outcomes, some genomic signatures may well be more useful than others, and our method may be helpful in providing a level of consistency across datasets, not just in prognostic stratification power, but also in association of the identified phenotype with molecular features and biological processes.

As the IRA is based on the refinement of training class labels, it is well-suited for use in settings where training classes are not a priori obvious, such as determination of relative benefit of one treatment over another or when the gold-standard endpoints are TTE outcomes. It is also well-suited to problems where the training class labels are known in principle, but where they are missing for some instances or known to be noisy or only partially accurate. Examples in the medical setting where training labels are known to be noisy include histological subtyping and gene copy number measurement [37].

Many aspects of this iterative refinement process remain to be investigated. Here, in addition to the synthetic data investigations, we have studied two prognostic stratification problems in oncology using mRNA datasets. The methods have also previously been used with measurements of the circulating proteome [16, 17]. However, we have yet to understand how generic the convergence properties of the algorithm are and how much the feature space structure depends on the type of classification problem, the clinical setting, and the measurement data (e.g. RNA-seq, epigenetic data). We have implemented the IRA using two different classifier development paradigms and, as long as an unbiased classification can be provided for development set samples, the method is agnostic to the supervised method used for classification. Different paradigms may be better suited to particular kinds of classification task and also provide alternative avenues for guiding or steering the final classifier towards optimal performance for the desired task.

## Conclusions

The iterative refinement approach simultaneously refines training class labels and the associated classifier to facilitate the robust identification of endpoint-associated phenotypes in molecular datasets. Its consistent incorporation of the structure of the molecular data into the classifier helps to minimize overfitting and enable good generalization of classification and molecular phenotypes. The method also aids the reliable identification of biologically relevant features and elucidation of underlying biological processes. Hence, the iterative refinement paradigm provides many advantages when working with binary classification problems when training class labels are ambiguous or poorly defined.

## Methods

### Synthetic datasets

Synthetic data was used to define sets containing two molecular phenotypes. The datasets were composed of 1000 attributes. Attribute values were randomly selected for N_S_ samples from a multivariate normal distribution. Each one of first nine hundred attributes was uncorrelated with all other attributes and centered around 0 with a variance of 1. One hundred additional attributes were used to define the molecular phenotype. They were selected to be uncorrelated with the first 900 attributes and randomly correlated within the subset of 100 attributes. The correlations were established using the method of Numpacharoen and Atsawarungruangkit [38]. N_A_ samples were defined as phenotype A and N_B_ samples as phenotype B (N_A_ + N_B_=N_S_). The 100 phenotype-defining attributes were centered at 0 with variance 0.1 for phenotype A and centered at 2 with variance 1 for phenotype B. A survival time was randomly assigned to each sample from an exponential distribution. No censoring of survival was considered. Survival times for samples with phenotype B were reduced by a scaling factor dependent on the values of the 100 phenotype-defining attributes. The scaling factor was calculated as follows. First, a large reference dataset of 10,000 instances in phenotype B was generated. Principal component analysis (PCA) was then performed on the 100 phenotype-defining attributes of this reference dataset to identify the first principal component (PC). The projection of the attribute values of each instance on the first PC was calculated and the 5th and 95th percentiles (“lower cutoff” or l and “upper cutoff” or u) of these projections were determined. The definition of the first PC, u and l were used to define the scaling factor for the phenotype B instances in the classification datasets. The vector of values of the 100 phenotype-defining attributes was projected onto the reference first PC to yield the projection, p, of each instance and the scaling factor for each instance defined as 1 + α (p-l)/(u-l) if p > l and 1 if p ≤ l. Lastly, survival times associated with instances with phenotype B were then divided by this scaling factor, so that survival for phenotype B was shorter than that for phenotype A and the difference in survival between the phenotypes could be controlled by the parameter, α.

Simulations were used to assess the distribution of hazard ratios between true phenotypes and the concordance between the true phenotypes and the initial training class labels as defined by survival dichotomization within the synthetic dataset. The sampling distributions for synthetic datasets for size N_A_ = N_B_ = 60 were estimated for fixed α by generating 1000 dataset realizations and evaluating the desired metrics in each realization.

The synthetic datasets demonstrate a highly simplified model with each instance in one of only two survival-related molecular phenotypes. This allows investigation of how the IRA can improve identification of the known phenotypes and isolation of the set of the attributes that define them.

### mRNA cohorts

Two two-class classification problems were chosen to demonstrate the properties and application of the IRA in real world datasets. The first example is the prognostic stratification of patients with breast cancer undergoing surgery and adjuvant chemotherapy. The second example is the stratification of patients with lymphoma treated with chemotherapy into good and poor prognosis groups. Both examples use publicly available mRNA expression datasets including TTE endpoints.

#### Breast Cancer

Two mRNA expression datasets generated from tissue samples collected from patients with non-metastatic breast cancer at time of surgery were available. The datasets were accessed from the supplementary materials associated with Venet et al. [35]. The NKI set was collected from 295 patients and consists of the expression of 13,108 genes [39], while the Loi set consists of expression values for 17,585 genes profiled for 380 patients [40]. The Loi set can also be accessed as GSE6532 within the Gene Expression Omnibus. The two sample sets had 12,770 genes in common and our investigations use only these as features for classification. Profiling had been carried out using Affymetrix U133 microarrays. The ComBat (http://www.bu.edu/jlab/wp-assets/ComBat/Abstract.html) platform [41] was used to make the two datasets compatible. ComBat is an empirical Bayes method that standardizes across datasets by trying to set the expression of each gene to a mean of 0 and a standard deviation of one. The NKI set was split into a development set of 148 samples and an internal validation set of 147 samples. The Loi set was used as an independent validation set.

#### Lymphoma

A gene expression profiling dataset of samples from 181 patients with diffuse large-B-cell lymphoma treated with CHOP (Cyclophosphamide, Hydroxydaunorubicin, Oncovin and Prednisone) chemotherapy was selected from the Gene Expression Omnibus (GSE10846). Profiling had been carried out using Affymetrix U133 plus 2.0 microarrays to yield expression data on 21,024 genes (features) and overall survival data were available [42, 43]. The cohort was split into a development set (*N* = 91) and an internal validation set (*N* = 90).

### Evaluation approach

For the synthetic datasets with known phenotypes, performance of the IRA was primarily evaluated by the ability of the developed classifiers to identify the true phenotypes. In particular this was assessed by concordance of classifier-defined phenotype with the true phenotype in both development set and validation sets. Out-of-bag estimates were used to obtain reliable instance classifications within the development sets. Hazard ratios between classifier-defined phenotypes were also evaluated and studied as the iterative refinement converged in both development and validation sets.

Real world datasets may contain many coexisting molecular phenotypes, and these are not known a priori. Hence, the performance of the IRA and the classifiers developed within it were assessed via the Cox proportional hazard ratio between classifier-defined phenotypes for the relevant TTE endpoint (OS for lymphoma and RFS for breast cancer) in both development set (by out-of-bag estimates) and the validation sets.

### The iterative refinement approach

**Fig. 8:**
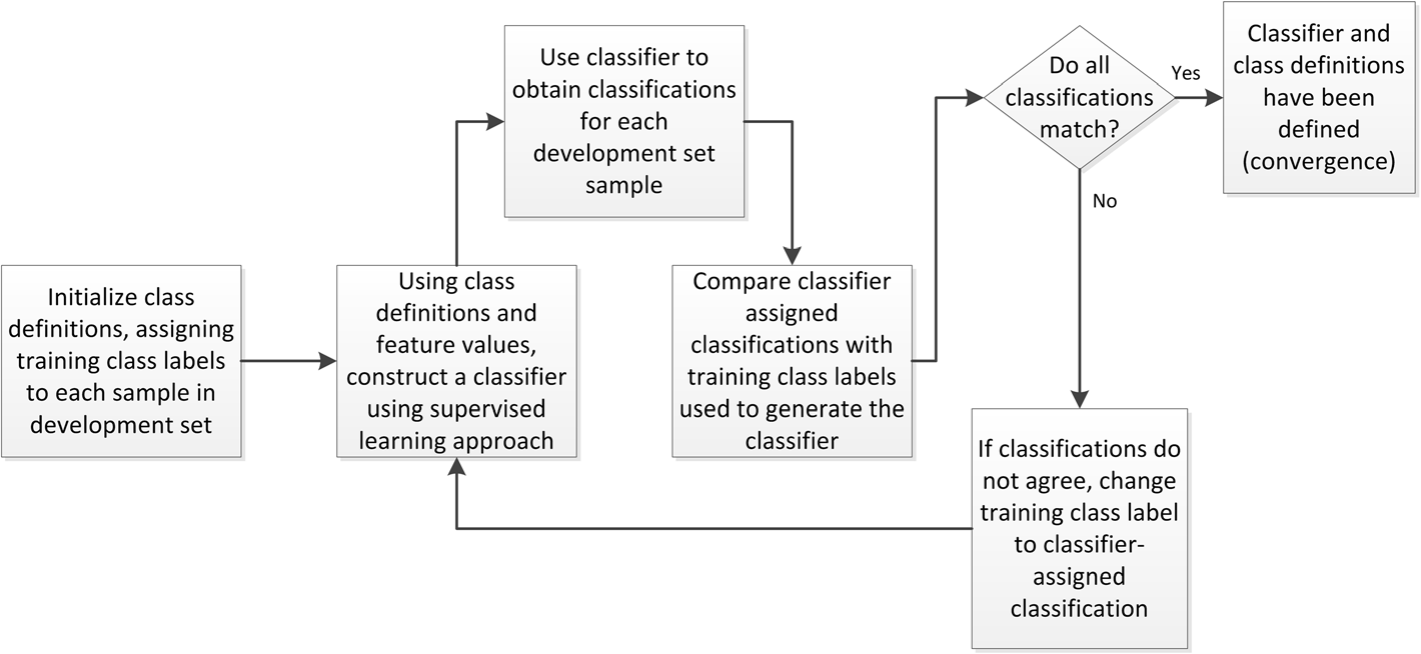
Schema showing the process of simultaneous refinement of training class labels and classifier

**Fig. 9:**
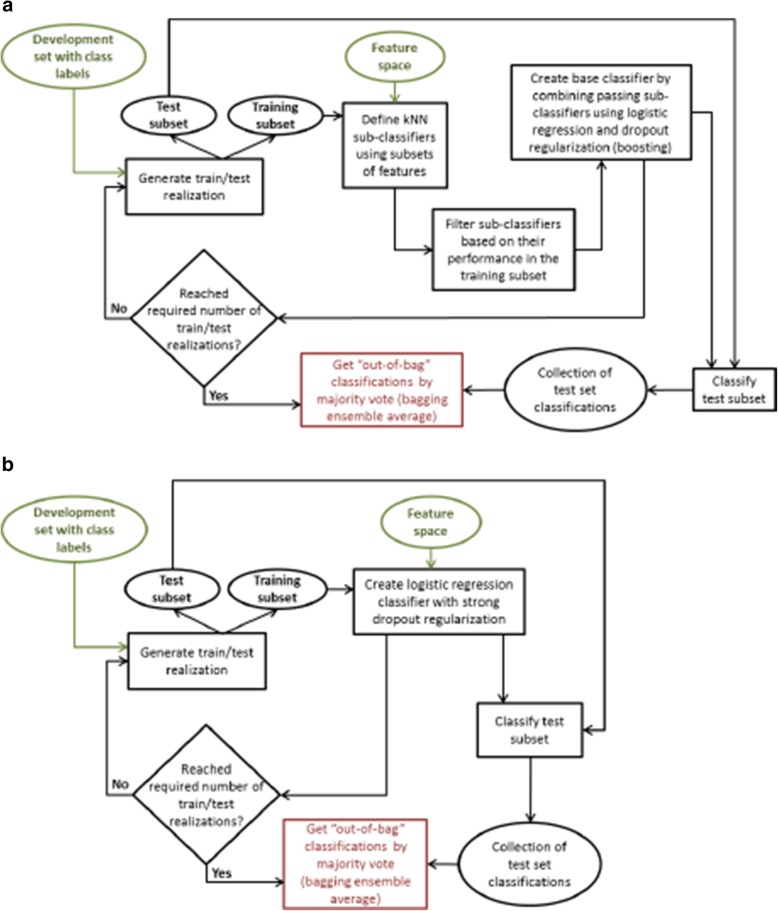
a:  Architecture of dropout-regularized combination classifier, b: Architecture of bagged logistic regression classifier

**Fig. 10:**
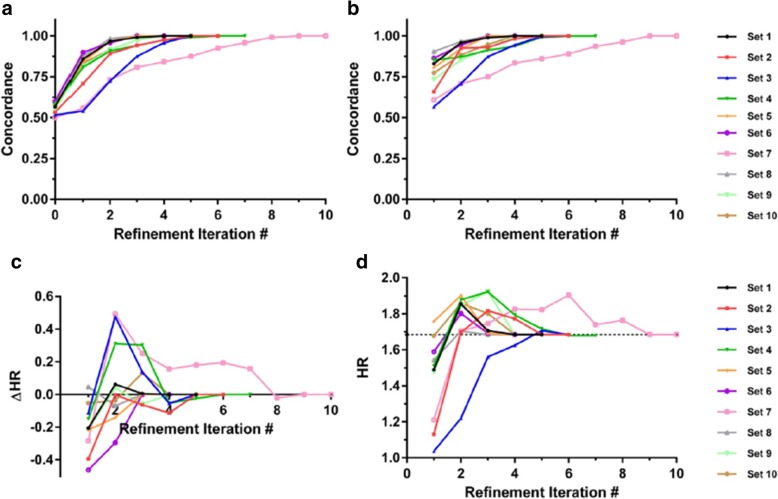
Development sets results for synthetic datasets with ratio of phenotype A:B of 30:90 and α = 1. For each development set realization the IRA was applied. At each refinement iteration, the classifiers were applied to their development set realization and the independent validation set. Concordance of classifier-derived phenotype with true phenotype is shown for (**a**) the ten development set realizations, and (**b**) the validation set, for all ten development set realizations as a function of refinement iteration. The difference between the hazard ratio for classifier-derived phenotypes and the hazard ratio for phenotype A vs phenotype B in the development sets, ∆HR, is shown in (**c**) as a function of refinement iteration. The hazard ratios for classifier-derived phenotypes in the validation set as a function of refinement iteration are shown in (d). The value of the hazard ratio in the validation set for phenotype A vs B (HR = 1.68) is indicated by the dashed line

**Fig. 11:**
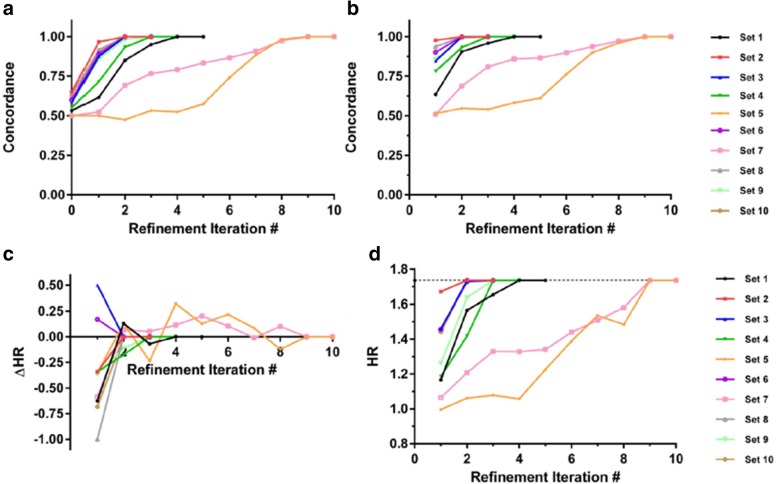
Development sets results for synthetic datasets with ratio of phenotype A:B of 90:30 and α = 1. For each development set realization the IRA was applied. At each refinement iteration, the classifiers were applied to their development set realization and the independent validation set. Concordance of classifier-derived phenotype with true phenotype is shown for (**a**) the ten development set realizations, and (**b**) the validation set, for all ten development set realizations as a function of refinement iteration. The difference between the hazard ratio for classifier-derived phenotypes and the hazard ratio for phenotype A vs phenotype B in the development sets, ∆HR, is shown in (**c**) as a function of refinement iteration. The hazard ratios for classifier-derived phenotypes in the validation set as a function of refinement iteration are shown in (**d**). The value of the hazard ratio in the validation set for phenotype A vs B (HR = 1.74) is indicated by the dashed line

**Fig. 12:**
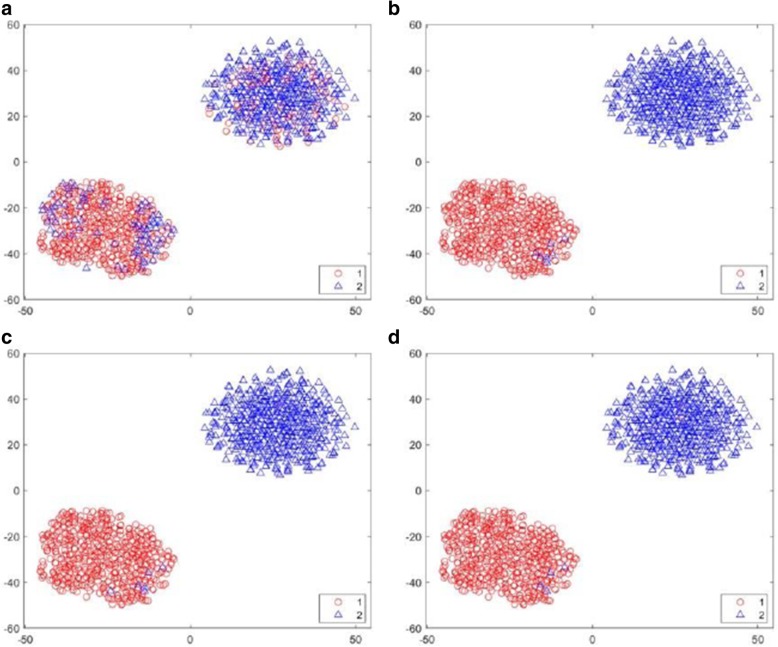
t-SNE plots for the initial training class labels and the classifications at each refinement iteration. Results are shown for the 1000 instance validation set (α = 1, N_A_ = N_B_ = 60) for the IRA using development set realization 1 for **a**) initial training class labels, **b**) classifier-derived phenotypes using initial training class labels (refinement iteration 1), **c**) classifier-derived phenotypes using training class labels from refinement iteration 1 (“refinement iteration 2”), **d**) classifier-derived phenotypes at convergence at refinement iteration 3. x and y axes show arbitrary scales of the two t-SNE components

**Fig. 13:**
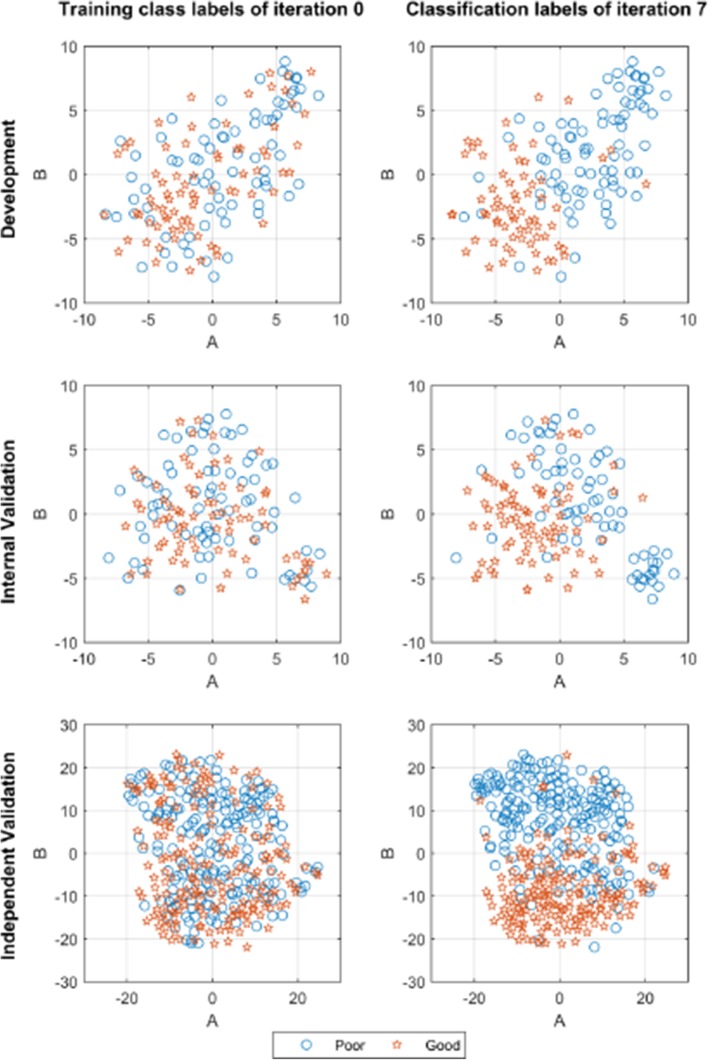
t-SNE plots for the initial training class labels and the classifications at refinement iteration 7 for the development set, the internal validation set, and the independent validation set

**Fig. 14:**
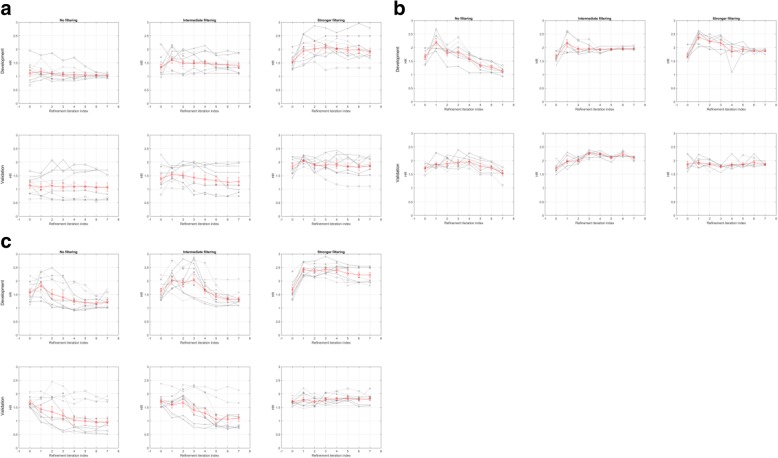
Hazard ratio as a function of refinement iteration for the three filtering settings for the individual initial condition realizations (grey) and their average (red) for: **a** – random initial conditions, **b** – TTE median-based initial conditions, **c** – 10% noise initial conditions, **d** – 20% noise initial conditions. Error bars show standard error

The methodology of iterative simultaneous refinement of training class labels and classifier is illustrated in Fig. 8.

The aim is to create a self-consistent system of classifier and class definitions. An initial set of class definitions for the development set is created by assigning each instance to one of the two training classes (e.g., good/bad prognosis, benefit/no benefit). Using these initial training class labels, a classifier is constructed using some chosen classification procedure. Any supervised learning scheme could be used as long as it provides unbiased classifications for samples that are used in development. This could be achieved, for example, by using a bagged approach to classifier development [19] and taking out-of-bag estimates [20] for classification of the instances used in training. The classifier created with the initial training class label assignment is used to classify the development set instances. Some of the resulting classifications will match the initial training class labels used for classifier development and some will not. The classifications produced by the classifier constitute a new set of training class labels for the development sample set with which a new classifier can be trained in the next refinement iteration. This is iterated until the process converges to produce a classifier that reproduces the class labels that are used to generate it or until a previously set maximum number of refinement iterations have been completed. This approach is similar in spirit to the Yarowsky algorithm in computational linguistics [44], and it can also be considered as a clustering method.

In general, the resulting classifier and classifier-defined phenotype depend on the initial conditions of the refinement process, i.e., the initial choice of development set training class labels, and may or may not prove useful for a particular practical application. To maintain the putative advantages of the process while steering it towards a final classifier and phenotype more likely to fulfill a specific performance goal, one can make use of outcome data. This can be done via two main approaches: choice of the initial training class labels and tuning of the classifier development within the refinement iterations. If the goal is to produce a good prognostic classifier, it is reasonable to start the iterative process not from a random initial condition, where the two classes are likely to have similar outcomes, but rather from a point where the two assigned sets of class-labeled instances have different outcomes. For example, instances associated with patients with the longest survival can be initially assigned to the good prognosis class and those with the shortest survival to the poor prognosis class. Instances which cannot be unambiguously assigned within this initial scheme, e.g., because of censoring of TTE outcomes, can be assigned randomly. The options available for integration of endpoint data into the classifier development step of the iterative refinement process (tuning) depend on the choice of classification paradigm. For classifiers which incorporate boosting, i.e., the combination of sub-classifiers to produce a final classifier with as good or better performance than the individual sub-classifiers [45], the endpoint data can be used to filter or prune the sub-classifier pool to leave only those that have a minimal level of performance with respect to a metric defined by for the endpoint. For example, if the aim is to stratify patients according to survival, we can test the ability of the sub-classifiers to carry out this task on the training set, or another sample set, by calculating the survival hazard ratio between patients assigned by each sub-classifier to the good and poor prognosis classes. Only sub-classifiers that demonstrate a specified level of performance for this task would be combined in the boosting step of the classifier development process, while the other sub-classifiers would be discarded. Note that if the sub-classifiers provide a biased classification of the data, this can be compensated for by choice of a higher threshold for filtering than might otherwise be deemed acceptable. Hence unbiased classification of the training set by the sub-classifiers is not an essential element.

Here we demonstrate each of the above scenarios. To examine the effect of incorporating TTE data into classifier development via choice of TTE-based initial training class label assignment together with feature selection during classifier development, we used bagged logistic regression as the classifier development paradigm. The method included strong dropout regularization to minimize overfitting in the setting of many more attributes than training instances. Full details can be found in Appendix A. This approach was used for the breast cancer datasets. The process was started with initial training class labels determined by dichotomization of RFS, and for each refinement iteration only the 100 attributes most strongly associated with the training class labels, as determined by a t-test, were selected for inclusion in classifier development.

To compare results between the fully unsupervised case, starting from randomly selected initial training class labels, initial training class labels chosen according to the TTE data, use of TTE data within classifier training, and combinations of these options, we used a hierarchical, dropout-regularized combination approach to classifier development that incorporates boosting, bagging and strong regularization. This DRC paradigm was also used for the synthetic data investigations. This method was designed for use in settings with many attributes and relatively few training instances, and it has been previously used in the field of personalized medicine test development [16–18]. More details can be found in Appendix A. This approach does not require any feature selection and time-to-event data can be easily incorporated to filter, with variable strength, the k-nearest neighbor sub-classifiers created using subsets of the feature space prior to their combination under strong regularization via dropout. Specifically, each of the sub-classifiers is applied to the training set to classify each training set instance, splitting the training set into the two classification groups. The hazard ratio for a TTE endpoint between these two groups is calculated using Cox proportional hazards methods and must exceed a chosen threshold for the sub-classifier to be included in the boosted combination. Sub-classifiers not meeting the criterion are discarded. The threshold can be tuned to study the effect of stronger or weaker filtering.

Note that no optimization or tuning of the parameters of the classifier development algorithms was performed and the goal of these studies was to assess the benefits of the IRA, not to compare between different classifier development paradigms. Hence, no optimization of classifier algorithm parameters was performed based on results from the IRA. Parameter values (see Appendix A) were defined from prior experience at the beginning of the study and held fixed throughout.

### Software

Software implementing the methods presented in this study is available at https://bitbucket.org/ in the BiodesixDxCortex2 repository.

## Availability and requirements

Project Name: BiodesixDxCortex2.

Project Home Page: https://bitbucket.org/diagnosticcortex/biodesixdxcortex2

Operating System(s): Windows Server 2012 R2 Standard.

Programming Language(s): Matlab R2017a.

License: New (3-clause) BSD license, https://en.wikipedia.org/wiki/BSD_licenses#3-clause

Data: The dataset supporting the conclusions of this article is available in the supplementary materials associated with Venet et al. [35] at 10.1371/journal.pcbi.1002240.s001 and in the Gene Expression Omnibus under GSE10846 and GSE6532. The datasets after any preprocessing prior to classifier development are available in the BiodesixDxCortex2 repository at https://bitbucket.org/diagnosticcortex/biodesixdxcortex2.

## Appendix

### Appendix A: Methods

**Table 2:** Parameters used with the dropout regularized classifier for the synthetic data investigation

Method	Parameter	Value(s)
DRC classifier (applied to the synthetic data)	k (kNN sub-classifiers)	9
	Subsets of features used in the sub-classifiers	Singles
	Sub-classifier filtering criteria	Survival HR between the two classification groups
	Sub-classifier filtering range applied to the training set	[1.5; 10.0]
	Number of dropout iterations (in the boosting step)	15,000
	Number of sub-classifiers kept in each dropout iteration	4
	Number of training / test realizations	325
	Number of samples included in the training subset, for each class	$2/3\times {N}_{\mathsf{S}}$, where ${N}_{\mathsf{S}}$ is the number of samples in the smaller class. Remainder samples assigned to the test subset
	Maximum number of refinement iterations	10

**Table 3:** Parameters used with the logistic regression classifier for the breast cancer application

Method	Parameter	Value(s)
Bagged logistic regression (applied to the breast cancer data set)	Number of features used in training (selected by t-test)	100
	Number of features used in each dropout iteration	1
	Number of dropout iterations (in the boosting step)	5000
	Number of training / test realizations	101
	Number of samples included in the training subset, for each class	$2/3\times {N}_{\mathsf{S}}$, where ${N}_{\mathsf{S}}$ is the number of samples in the smaller class. Remainder samples assigned to the test subset
	Maximum number of refinement iterations	10 (converged at iteration 8)

**Table 4:** Parameters used with the dropout regularized combination classifier for the lymphoma application

Method	Parameter	Value(s)
DRC classifier (applied to the lymphoma data set)	k (kNN sub-classifiers)	9
	Subsets of features used in the sub-classifiers	Singles
	Sub-classifier filtering criteria	OS HR between the two classification groups
	Sub-classifier filtering range applied to the training set	[0.0; 100.0] (no filtering), [1.3; 100.0] (intermediate filtering) and [2.0; 100.0] (strong filtering)
	Number of dropout iterations (in the boosting step)	100,000
	Number of sub-classifiers kept in each dropout iteration	10
	Number of training / test realizations	375
	Number of samples included in the training subset, for each class	$2/3\times {N}_{\mathsf{S}}$, where ${N}_{\mathsf{S}}$ is the number of samples in the smaller class. Remainder samples assigned to the test subset
	Maximum number of refinement iterations	8

### Appendix B: Results

1. **Synthetic Data**

2. **Breast cancer dataset**
3. **Lymphoma dataset**

## Abbreviations

∆HR: the difference between the hazard ratio for survival between the classifier-defined phenotypes and the hazard ratio for survival between the true phenotypes, A and B
HER2: human epidermal growth factor receptor 2
HR: hazard ratio
IC: initial condition
IQR: inter-quartile range
IRA: iterative refinement approach
mRNA: messenger Ribonucleic Acid
OS: overall survival
PH: proportional hazards
RFS: recurrence-free survival
TTE: time-to-event
